# Effective high-throughput isolation of enriched platelets and circulating pro-angiogenic cells to accelerate skin-wound healing

**DOI:** 10.1007/s00018-022-04284-4

**Published:** 2022-04-26

**Authors:** Silvia Erratico, Marzia Belicchi, Mirella Meregalli, Dario Di Silvestre, Luana Tripodi, Antonella De Palma, Rebecca Jones, Emanuele Ferrari, Laura Porretti, Elena Trombetta, Giorgio R. Merlo, Pierluigi Mauri, Yvan Torrente

**Affiliations:** 1 Novystem Spa, viale Piave 21, 20129 Milan, Italy; 2 Unit of Neurology, Stem Cell Laboratory, Department of Pathophysiology and Transplantation, Universitá degli Studi di Milano, Fondazione IRCCS Ca’ Granda Ospedale Maggiore Policlinico, Centro Dino Ferrari, via Francesco Sforza 35, 20122 Milan, Italy; 3 Institute of Technologies in Biomedicine, National Research Council (ITB-CNR), Via Fratelli Cervi, 93, Segrate, 20090 Milan, Italy; 4 Department of Molecular Biotechnology and Health Science, University of Torino, Via Nizza 52, 10126 Turin, Italy; 5 Flow Cytometry Service, Fondazione IRCCS Ca’ Granda Ospedale Maggiore Policlinico, via Francesco Sforza 35, 20122 Milan, Italy

**Keywords:** Epithelialization, PRP, Angiogenic potential, Skin remodeling, Proteomics

## Abstract

Delayed wound healing and chronic skin lesions represent a major health problem. Over the past years, growth factors mediated by platelet-rich plasma (PRP) and cell-based therapies were developed as effective and affordable treatment able to improve wound healing capacity. We have advanced existing concepts to develop a highly efficient high-throughput protocol with proven application for the isolation of PRP and pro-angiogenic cells (Angio^PRP^). This protocol outlines the effectiveness of Angio^PRP^ in promoting the critical healing process including wound closure, re-epithelialization, granulation tissue growth, and blood vessel regeneration. We coupled this effect with normalization of mechanical properties of rescued mouse wounds, which is sustained by a correct arrangement of elastin and collagen fibers. Proteomic analysis of treated wounds demonstrated a fingerprint of Angio^PRP^ based on the up-regulation of detoxification pathway of glutathione metabolism, correlated to a decrease in inflammatory response. Overall, these results have enabled us to provide a framework for how Angio^PRP^ supports wound healing, opening avenues for further clinical advances.

**Supplementary Information:**

The online version contains supplementary material available at 10.1007/s00018-022-04284-4.

## Introduction

Wound healing is a dynamic and orchestrated sequence of events requiring the interaction of soluble mediators, blood cells and extracellular matrix that result in the restoration of skin integrity and homeostasis [1]. Wound repair proceeds in three overlapping and functionally distinct phases characterized first by infiltration of neutrophils and macrophages, [2] followed by angiogenesis, fibroblasts and keratinocytes proliferation [3] that allows granulation tissue formation and extracellular matrix remodeling [4, 5]. An interruption in the normal wound healing process can lead to the development of non-healing chronic wounds, a typical complication of several diseases, such as foot ulcer from diabetes and pressure ulcer resulting from spinal cord injuries [6]. As wound healing impairment represents a major health problem, the complexity of cell and molecular events required for appropriate repair constitute a major research focus [7, 8]. In this regard, different dressing and ointments, such as hydrocolloids, alginates, foams, sulfadiazine silver patches, and honey gauzes, have been described to promote chronic wound healing [9]. Nevertheless, the systematic review [10] of local interventions do not support conclusive evidences for ulcer healing. Other evidences suggest that hyperbaric oxygen and negative pressure wound therapy systems can induce and accelerate wound healing [11]; however these interventions are limited by reduced availability, patients’ intolerance and high costs. For extensive wounds, a variety of skin substitutes are available, that can be classified by origin (allogenic, xenogeneic, and autologous), composition (dermal, epidermal or both components) or timing (durable or temporary substitutes) [12, 13]. The ideal skin substitute performs the functions of skin, while being cost-effective, widely available, and easy to apply [14]. Platelet-rich plasma (PRP) has shown promising experimental and clinical results in chronic wound. Moreover, application of PRP has been demonstrated to be effective in soft tissue reconstruction [15, 16], bone reconstruction [17, 18] and hair regrowth [19–22]. The addition of bioactive excipients, both natural as fat graft and synthetic (i.e. hyaluronic acid, 3D collagen scaffolds) has also been suggested to accelerate endothelial, epithelial and epidermal regeneration of PRP [15, 17, 23, 24]. The major families of growth factors that are released from PRP and are involved in wound healing includes factors that stimulates fibroblasts to secrete collagenases during the remodeling phase and encourages keratinocyte and fibroblast proliferation [25]. Increased rates of cell proliferation and cell migration have been associated with the upregulation of different cell-cycle-regulatory proteins and PI3K/AKT/NF-kB signaling pathways [26, 27]. Although PRP is a source of growth factors, and consequently has mitogenic, angiogenic, and chemotactic properties, representing an interesting alternative adjunctive treatment for acute and chronic wounds, PRP is far from standardized and the most effective way of application has yet to be defined. Further, commercial PRP separation systems vary widely regarding the harvest and concentration of various PRP substances. Chronic wounds also occur with complications of impaired angiogenesis [28, 29] and transplantation of endothelial progenitor cells (EPCs) has demonstrated promising results in wound healing [30]. EPCs are bone marrow mononuclear progenitor cell that were first discovered as circulating cells in peripheral blood [31, 32] and characterized for their capacity to increase angiogenesis and vascularization by secreting growth factors and cytokines in damaged tissues [33]. Circulating EPCs displayed specific cell surface markers such as CD45 to identify their hematological origin in combination with different endothelial surface markers, such as CD31, CD144 or CD146 [34, 35]. Recent studies suggest that angiogenic T cells (Tangs) may regulate EPC function [36–38]. Tang express CD31 as well as the receptor for stromal derived factor 1 (CD184) [38] and promote the formation of new blood vessels and endothelial repair by stimulating the function of EPC [38].

Based on the above, it is reasonable to speculate that a combination of PRP and pro-angiogenic cells could exert a synergistic positive effect on keratinocyte proliferation and angiogenesis accelerating wound healing. Here we describe an optimized single-use sterile closed system for the high-throughput isolation of human PRP and circulating EPCs and Tangs (hereafter named Angio^PRP^) that is highly reliable and effective in enhancing the wound healing process. In pursuit of translational outcomes, we developed a procedure for applying Angio^PRP^ in vitro in human skin and in vivo in mouse dorsal skin excisional wounds. For comparison purposes, we applied PRP and Hyalomatrix, a dermal matrix which is the state-of-the-art treatment currently used for patients with deep wounds. We found that Angio^PRP^ promotes angiogenesis and increases the wound healing of damaged organotypic human skin. In a second extent, we demonstrated that Angio^PRP^ reduced inflammation and promoted neo-angiogenesis throughout the repair of skin mouse wounds. The regenerative pathways of Angio^PRP^ on wound healing were investigated using proteomic and systems biology approaches, as previously reported [39]. Interestingly, Angio^PRP^ induced up-regulation of glutathione metabolism proteins involved in detoxification process, such as GSTZ1 e GSTT3. All these data recapitulate the regenerative outcomes of Angio^PRP^ in skin wounds.

## Results

### Angio^PRP^ is prevalently enriched of platelets and peripheral CD45+/CD31+/CD34− blood cells with Tang and EPC features

**Fig. 1:**
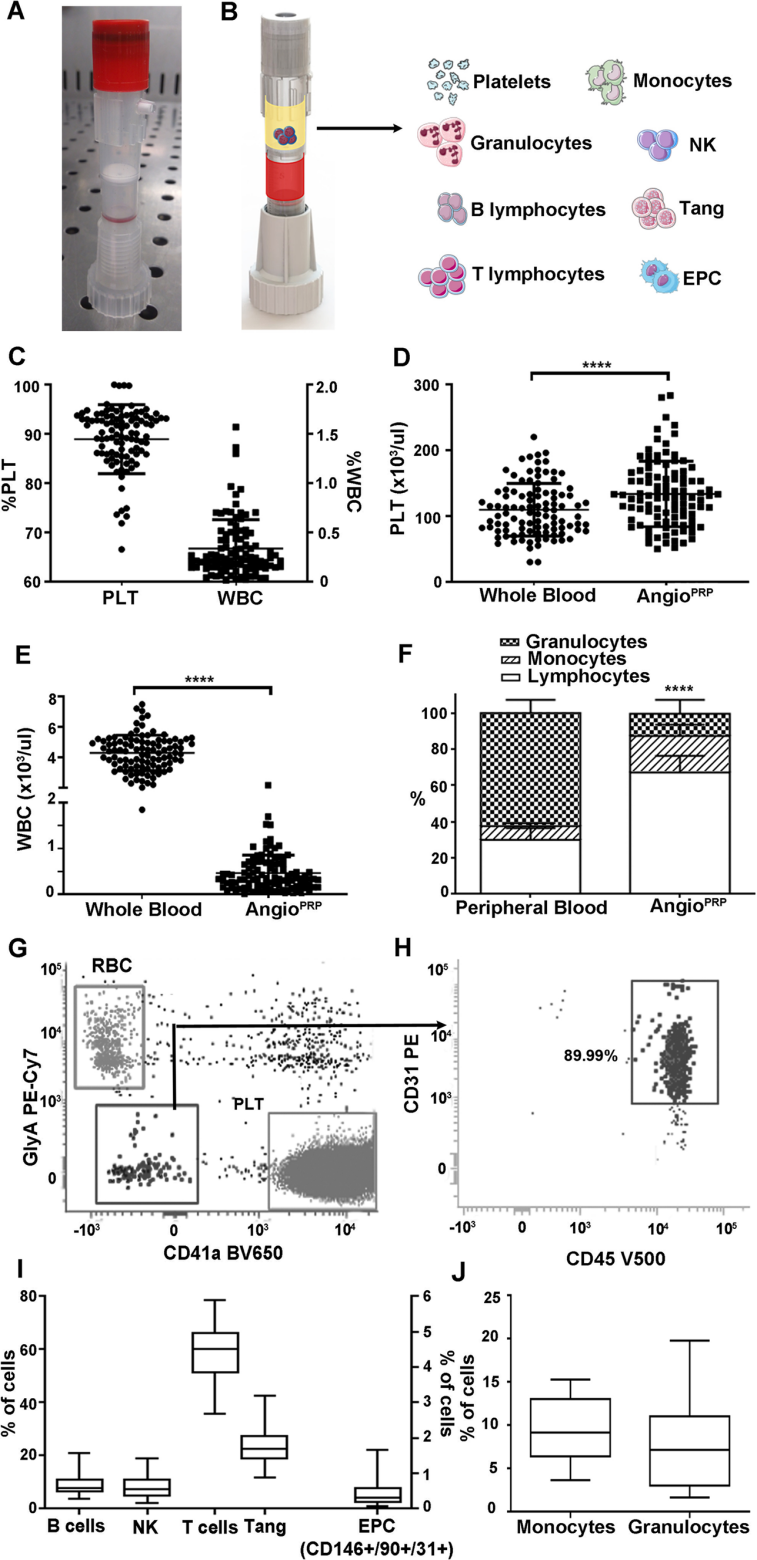
Angio^PRP^ in vitro characterization. **A** Picture of the Sep4Angio™ device. **B** Schematic overview of different components of Angio^PRP^ product obtained by Sep4Angio™ device. **C** Product composition, expressed as percentage of platelets and cells present in Angio^PRP^ (scale for platelets on left *y*-axis and for cells on right *y*-axis respectively). Analysis of platelets enrichment (**D**) and WBC reduction (**E**) in Angio^PRP^, compared to original whole blood (paired *t* test, ****p < 0.0001). **F** Distribution of white blood cells (granulocytes, monocytes and lymphocytes percentages) in Angio^PRP^, compared to whole blood leukocyte formula (paired *t*-test, ****p < 0,0001 for all the 3 parameters of the formula respectively). **G** Phenotypic characterization by flow cytometry of cellular component of Angio^PRP^. **H** Gated blood cells were characterized by CD31 and CD45 expression. **I** Flow cytometry quantification of B cells, NKs, T cells and Tang T cell sub-fraction (on left *y*-axis), and CD146+/90+/31+ EPC-like cells (on right *y*-axis) of Angio^PRP^. **J** Flow cytometry quantification of monocyte and granulocyte subpopulations range present in Angio^PRP^

We fabricated a single-use sterile closed system (Sep4Angio™) based on a collecting tube with an inert porous membrane of high-grade polyethylene, a rubber stopper to insert 2.5 ml of peripheral blood and a ring nut to adjust the plasma phase volume above the membrane after centrifugation (Fig. 1A, B). Cell Coulter counter analysis of the product obtained from peripheral blood separated through Sep4Angio™ revealed that Angio^PRP^ is composed mainly by platelets (88.92 ± 7.001%) with a low amount of white blood cells (WBCs) (0.34 ± 0.29%) (Fig. 1C). The platelets concentration was significantly increased in Angio^PRP^ compared to the whole-blood before preparation (133.6 ± 49.78 × 10^3^ instead of 109.6 ± 39.95 × 10^3^ platelets/µl, *p* < 0.0001) (Fig. 1D), while WBCs were significantly decreased (0.47 ± 0.39 × 10^3^ instead of 4.27 ± 1.17 × 10^3^ WBC/µl, Fig. 1E) (*n* = 101; *p* < 0.0001). Moreover, Coulter counter analysis demonstrated that Angio^PRP^ was significantly enriched of lymphocytes compared to the whole-blood hold before preparation (67.15 ± 9.25% for Angio^PRP^ and 29.98 ± 6.52% for whole blood, *n* = 25, *p* < 0.0001), while the granulocyte population was severely reduced (12.32 ± 7.67% for Angio^PRP^ and 62.36 ± 7.38% for whole blood, *n* = 25, *p* < 0.0001) and monocytes partially increased (20.13 ± 6.30% for Angio^PRP^ and 7.69 ± 1.56% for whole blood, *n* = 25, *p* < 0.0001) (Fig. 1F). In these analyses, the cellular component of Angio^PRP^ was discriminated from the red blood cells and platelets using CD41a and Glycophorin A labeling (double-negative population in Fig. 1G); cytometric characterization of Angio^PRP^ highlighted the immuno-phenotype of double-negative CD41a and Glycophorin A cells as CD45+/CD31+/CD34− (89.99 ± 8.86%; *n* = 19, Fig. 1H). Among these cells, we identified distinct subpopulations of lymphocytes (58.89 ± 9.61% of T cells, 23.10 ± 6.13% of Tangs as T cell subpopulation with angiogenic potential and 8.98 ± 3.53% of B cells), CD146+/90+/31+ EPCs (0.41 ± 0.29%), natural killers (NKs, 8.10 ± 3.79%), monocytes (9.62 ± 4.21%) and granulocytes (9.01 ± 7.50%) (Fig. 1I, J). Cytometry analysis plots for the specific cell subpopulations are reported in Supplementary Fig. S1.

### Angio^PRP^ promotes angiogenesis and increases the wound healing of damaged organotypic human skin

**Fig. 2:**
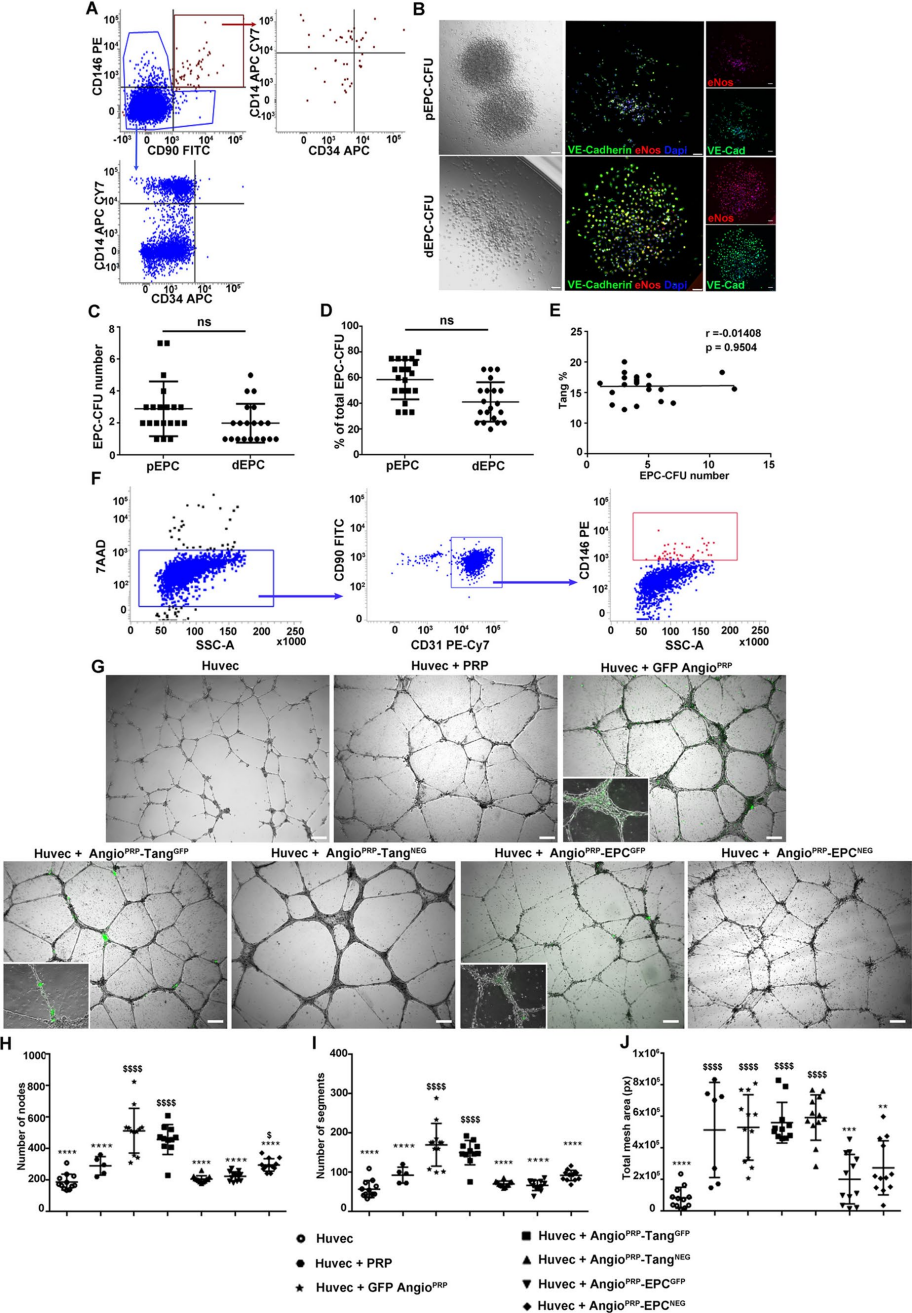
In vitro evaluation of Angio^PRP^ angiogenic potential. **A** Cytofluorimetric characterization of EPC and non-EPC fractions of Angio^PRP^. **B** Endothelial progenitor cell colony-forming assay (EPC-CFA) profile derived from Angio^PRP^; representative optical and immunofluorescence images of VE-cadherin (green) and eNOS (red) expression of small EPC colony-forming unit (pEPC-CFUs) and large dEPC-CFUs in primary EPC-CFA at × 10 magnification (scale bar = 250 µm). For fluorescence microscopy, nuclei were counterstained with DAPI and appeared in blue. **C**, **D** Quantification of pEPC and dEPC colonies obtained from Angio^PRP^ expressed as number of colonies and percentages on total colonies (differences are not statistically significant, ns). **E** Correlation by Spearman’s rank test between number of EPC-CFUs and percentage of Tang subpopulation from individual donor samples. **F** Flow cytometry characterization of EPC-CFUs obtained from Angio^PRP^ in EPC-CFA: 7AAD-negative gated cells were analyzed for expression of CD31, CD90 and CD146 markers. **G** Angiogenic assay performed in vitro on Matrigel in co-culture with Huvec for 24 h (scale bar = 250 µm). Quantifications of number of nodes (**H**), segments (**I**) and total mesh area (**J**) per field evaluated by ImageJ software (Angiogenesis analyzer) and expressed as mean ± SD (one-way ANOVA analysis of variance with Bonferroni correction; *comparison to GFP Angio^PRP^, ^$^comparison to Huvec; **p < 0.01; ****p < 0.0001)

**Fig. 3:**
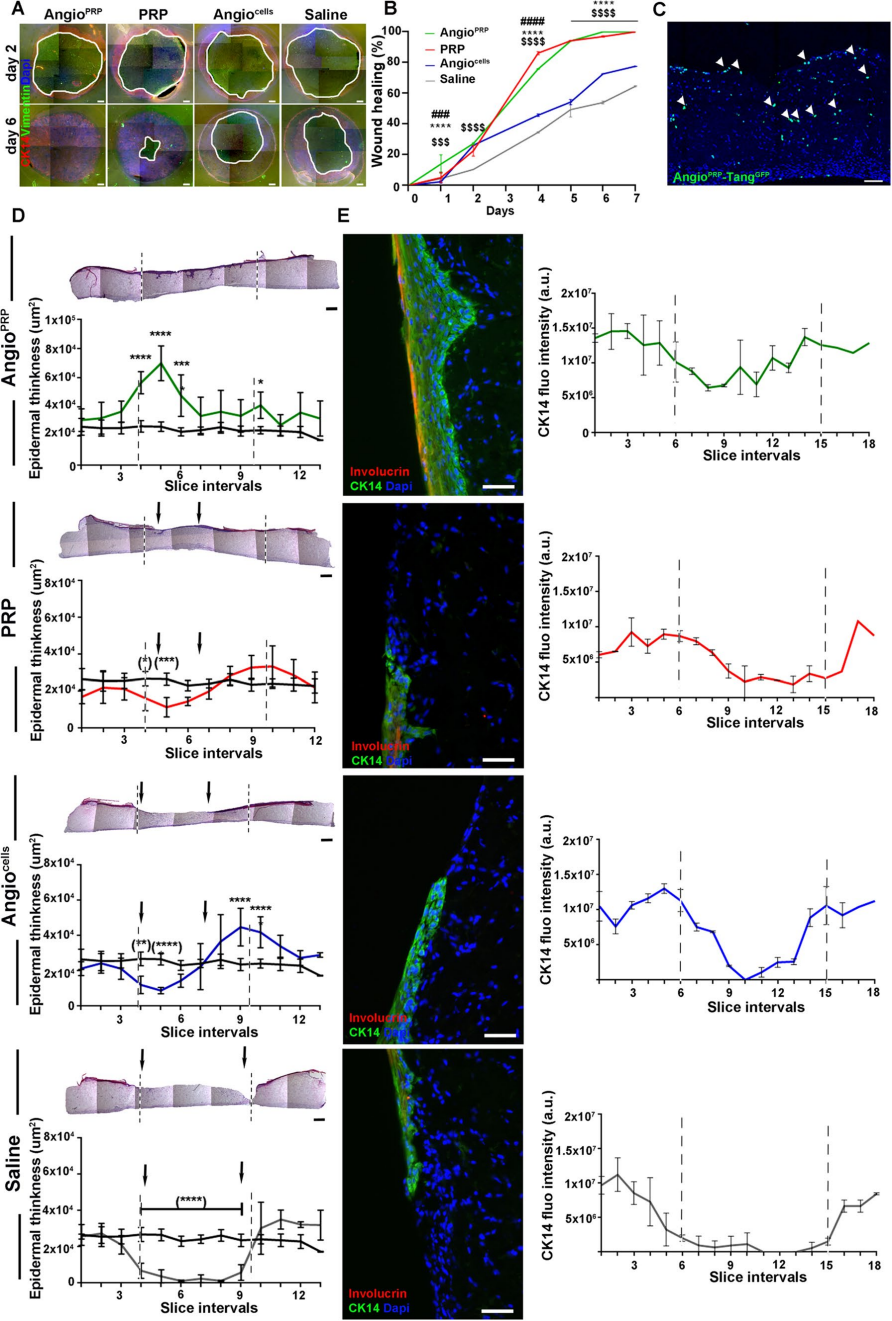
Angio^PRP^ validation and epidermal differentiation on organotypic culture. **A** Representative immunofluorescence staining images showing restitution of organotypic 3D skin tissue after wounding with a biopsy punch (5 mm in diameter). Epithelial cells shouldering the wound migrate to reseal the injured tissue 2 and 6 days after treatment with Angio^PRP^, PRP, Angio^cells^ and saline solution (scale bar = 500 µm). Migrating epithelial cells express cytokeratin 14 (red) and fibroblasts express vimentin (green); nuclei are stained with DAPI (blue). **B**Quantification of wound healing trend as percentage of wound area closure (two-way ANOVA analysis of variance with Bonferroni correction. *Angio^PRP^ vs PRP, ^$^Angio^PRP^ vs saline, ^#^Angio^PRP^ vs Angio^cells^. ***p < 0.001 ****p < 0.0001). **C** Wound treated with Angio^PRP^ including Tang subpopulation labelled with GFP (Angio^PRP^-Tang^GFP^) displayed GFP+ cells (arrows) at 3 DPI (scale bar = 100 µm) **D** Representative hematoxylin and eosin staining images of organotypic 3D skin section 6 days after treatment with Angio^PRP^, PRP, Angio^cells^ or saline solution (scale bar = 500 µm). Images were divided at 12 regular intervals (*x*-axis of relative graphics) representing the entire section and the epidermal thickness is quantified as area per interval. Epidermal thickness area quantification per interval of undamaged organotypic 3D skin is represented in black line as control. Dashed lines indicate the original wound boundary (5 mm-diameter excision); arrows show the lesion surface not yet re-epithelialized 7 days after wound. Data are presented as mean ± SD. Two-way analysis of variance (ANOVA) with Bonferroni correction (*p < 0.05, **p < 0.01, ***p < 0.001; ****p < 0.0001) was performed comparing control (black line) to the Angio^PRP^ (green line), PRP (red line), Angio^cells^ (blue line) and saline solution (grey line) epidermal thickness area. **E** Representative images of immunofluorescence staining for cytokeratin 14 (green) and involucrin (red) expression of organotypic 3D skin tissue section 7 days after Angio^PRP^, PRP, Angio^cells^ or saline solution treatments (scale bar = 75 µm). Nuclei are stained with DAPI (blue). Cytokeratin 14 fluorescence intensity is measured for every single interval along the tissue slice and reported as consecutive points on *x*-axis (green line for Angio^PRP^, red line for PRP, blue line for Angio^cells^ and grey line for saline). Dashed lines represent the original wound boundary. Data are presented as mean ± SD

In view of the presence of CD146+/90+/31+ EPC in Angio^PRP^, we evaluated the co-expression of CD34 and CD14 in EPC and non-EPC populations to better remark the presence of endothelial progenitor cells only in EPC (CD34+/14− 11.96 ± 4.94% for EPCs versus 0.23 ± 0.29% for non-EPCs; CD34+/14 + 13.85 ± 7.46% for EPCs versus 0.14 ± 0.09% for non-EPCs Fig. 2A). To assess angiogenic activities of Angio^PRP^, EPC-CFA (Endothelial Progenitor Cell—Colony Formation Assay) was used to monitor two different types of EPC-CFUs, pEPC-CFUs, and dEPC-CFUs, which comprised small and large cells respectively. pEPCs derive from relatively immature, highly proliferative EPCs, whereas dEPCs are relatively mature, differentiated, and able to promote EPC-mediated cell functions required for angiogenesis [40]. In addition, the expression of eNos and VE-Cadherin endothelial markers was higher in dEPCs compared to pEPCs (85.82 ± 2.08% vs 64.62 ± 3.49% for eNOS and 85.82 ± 1.23% vs 70.06 ± 2.4% for VE-Cadherin, Fig. 2B). Colony-forming cells (CFCs) from 5 × 10^4^ cells/dish of Angio^PRP^ generated 2.9 ± 1.7 pEPC and 2.0 ± 1.2 dEPC colonies per dish, corresponding to 58.67% and 41.33% of the total number of colonies (Fig. 2C, D). Since we found Tang subpopulation in Angio^PRP^ product, we evaluated whether Tang cells could have participated in EPC-CFA. The absence of a correlation between the number of EPC-CFUs observed and the percentage of Tang in individual samples confirmed that EPCs were the main cellular component involved in the angiogenesis process (Spearman’s correlation, *r* = − 0.01408, *p* = 0.9504, Fig. 2E). Cytofluorimetry of CFCs-recovered cells showed co-expression of CD90 and CD31 and partial expression of CD146 confirming their angiogenic phenotype (Fig. 2F). To better evaluate the ability to undergo angiogenesis, we also performed an in vitro tube formation assay testing HUVEC seeded on Matrigel with PRP, Angio^PRP^ labelled with GFP (GFP Angio^PRP^), Angio^PRP^ including Tang subpopulation labelled with GFP (Angio^PRP^-Tang^GFP^), Angio^PRP^ without Tang subpopulation (Angio^PRP^-Tang^NEG^), Angio^PRP^ including EPC subpopulation labelled with GFP (Angio^PRP^-EPC^GFP^) or Angio^PRP^ without EPC subpopulation (Angio^PRP^-EPC^NEG^). All experimental conditions displayed capillary-like tubular structures after 24 h (Fig. 2G). Quantitative analysis of capillary-like tubular structures showed significant increase of the total number of nodes per field in GFP Angio^PRP^ (514.5 ± 141.8) related to Huvec (189.3 ± 50.26 *p* < 0.0001) and Huvec + PRP (292 ± 58.71 *p* < 0.0001) (Fig. 2H). Similar results were obtained from the number of total segments per field (GFP Angio^PRP^: 170.3 ± 54.1; Huvec + PRP: 93 ± 20.40; Huvec: 150.6 ± 30.7 *p* < 0.0001) (Fig. 2I). Difference in total mesh area was statistically significant between GFP Angio^PRP^ and Huvec control only (5.32 ± 2.07 × 10^5^ pixels and 8.54 ± 6.63 × 10^4^ pixels *p* < 0.0001) (Fig. 2J). To validate the effects of Angio^PRP^ on skin lesions, we performed an in vitro evaluation on reconstructed human skin (EpiDerm FT). A 5 mm diameter circular lesion was performed on organotypic skin samples and different conditions were tested. Wound healing was monitored daily and measured as percentage of lesion area closed. Epithelial cells shouldering the wound migrate to reseal the injured tissue express cytokeratin 14, whereas fibroblasts from dermis express vimentin (Fig. 3A). The Angio^PRP^ showed a complete healing 6 days post injury (DPI), while the treatment with PRP took one more day to reach the same results (Fig. 3A). The Angio^cells^ and saline solution could not succeed the complete healing in one week (Fig. 3B). Wounds treated with Angio^PRP^-Tang^GFP^ displayed GFP+ cells at 3 DPI (Fig. 3C). The epidermal thickness was measured along the skin sections stained with hematoxylin and eosin and all conditions were compared to healthy skin. TheAngio^PRP^-treated skin at 7 DPI showed significantly higher epidermal thickness than untreated healthy skin (*p* < 0.0001), while treatment with PRP, Angio^cells^ and saline solution led to lower epidermal thickness than healthy skin (*p* < 0.0001) (Fig. 3D). Moreover, the Angio^PRP^ treatment induced the formation of differentiated epithelium as shown by the presence of both cornified (Involucrin) and spinous (Cytokeratins 14) layers, whereas Angio^cells^ and saline solution-treated skin showed a thin and incomplete epidermal layer (Fig. 3D). The quantification of cytokeratin 14 immunofluorescence along the skin slices confirmed a completely reconstructed epithelium only in Angio^PRP^-treated samples (Fig. 3E).

### Angio^PRP^ accelerates wound healing in mouse

**Fig. 4:**
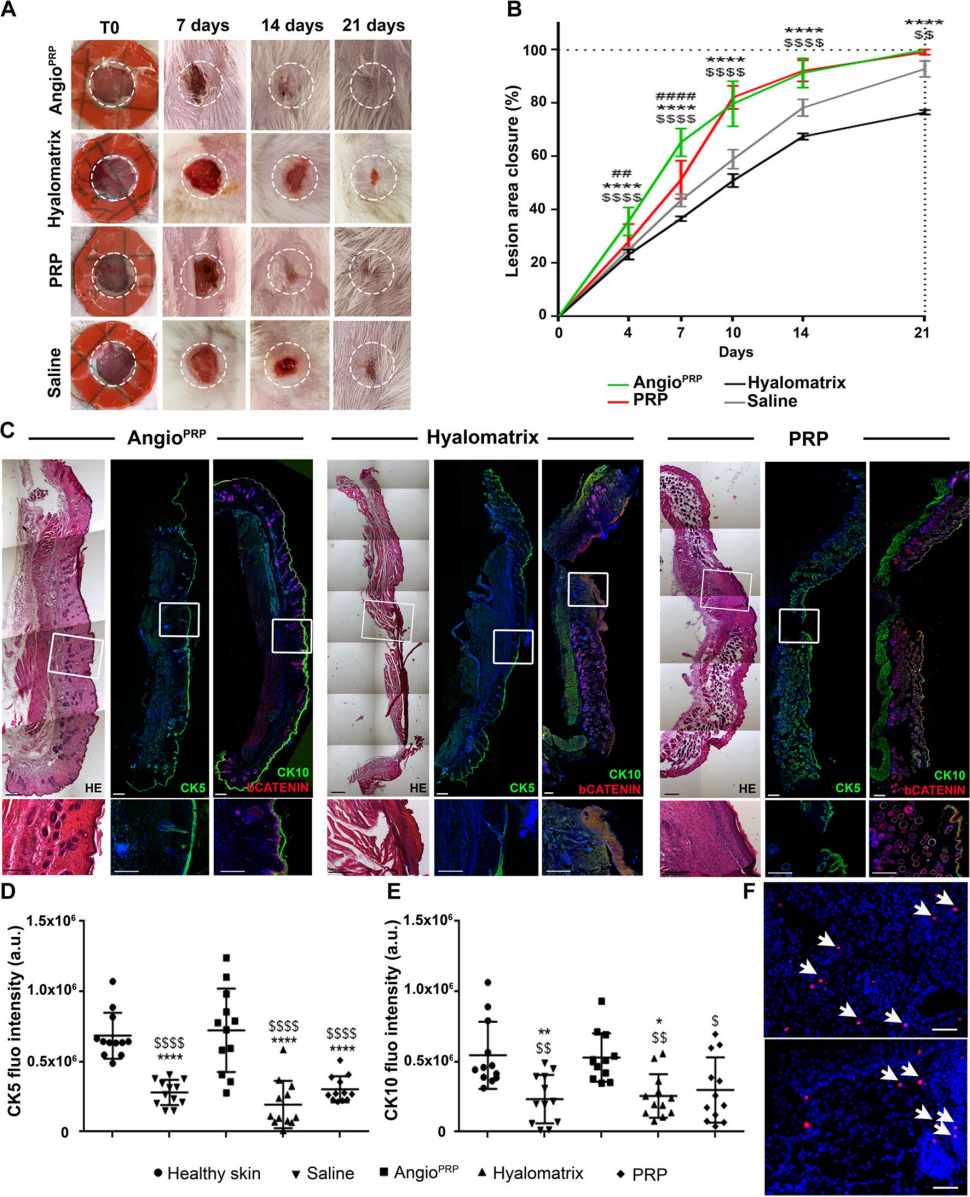
In vivo skin lesion closure and epidermal differentiation. **A** Representative images showing skin-wound closure of the NOD.Cg-Prkdc^Scid^/J at 0, 7, 14 and 21 days after wounding with a 5 mm biopsy punch (dashed white circle). **B** Wound closure rate is quantified as a ratio between the area measured and the area of the initial lesion at different time points (two-way analysis of variance (ANOVA) Bonferroni correction. *Angio^PRP^ vs Hyalomatrix, ^$^Angio^PRP^ vs saline, ^#^Angio^PRP^ vs PRP. **p < 0.01 ****p < 0.0001. **C** Hematoxylin and eosin histological reconstruction of skin section 21 days after Angio^PRP^, hyalomatrix or PRP treatment (left panels, scale bar = 500 µm). Immunofluorescence reconstructed images of cytokeratin 5 (CK5) staining for basal layer identification (central panels; scale bar = 250 µm) and cytokeratin 10 (CK10) and β-catenin staining for dermal–epidermal junction of Angio^PRP^, hyalomatrix and PRP-treated wounds at 21 DPI (right panels, scale bar = 250 µm). Frame magnifications are reported in the enlarged images (scale bar = 200 µm).) Fluorescence intensity quantification of images corresponding to cytokeratin 5 (**D**) and cytokeratin 10 (**E**) in the central wound area (mean ± SD for all the condition tested; one-way ANOVA analysis of variance with Bonferroni correction; ^$^comparison to healthy skin, *comparison to Angio^PRP^; *p < 0.05 **p < 0.01 ****p < 0.0001). **F** Representative immunofluorescence images of human nuclei-positive cells (HuNu) (arrows) in Angio^PRP^-treated wounds 7 and 21 days after injury (top panel 7 days, lower panel 21 days; scale bar = 50 µm)

**Fig. 5:**
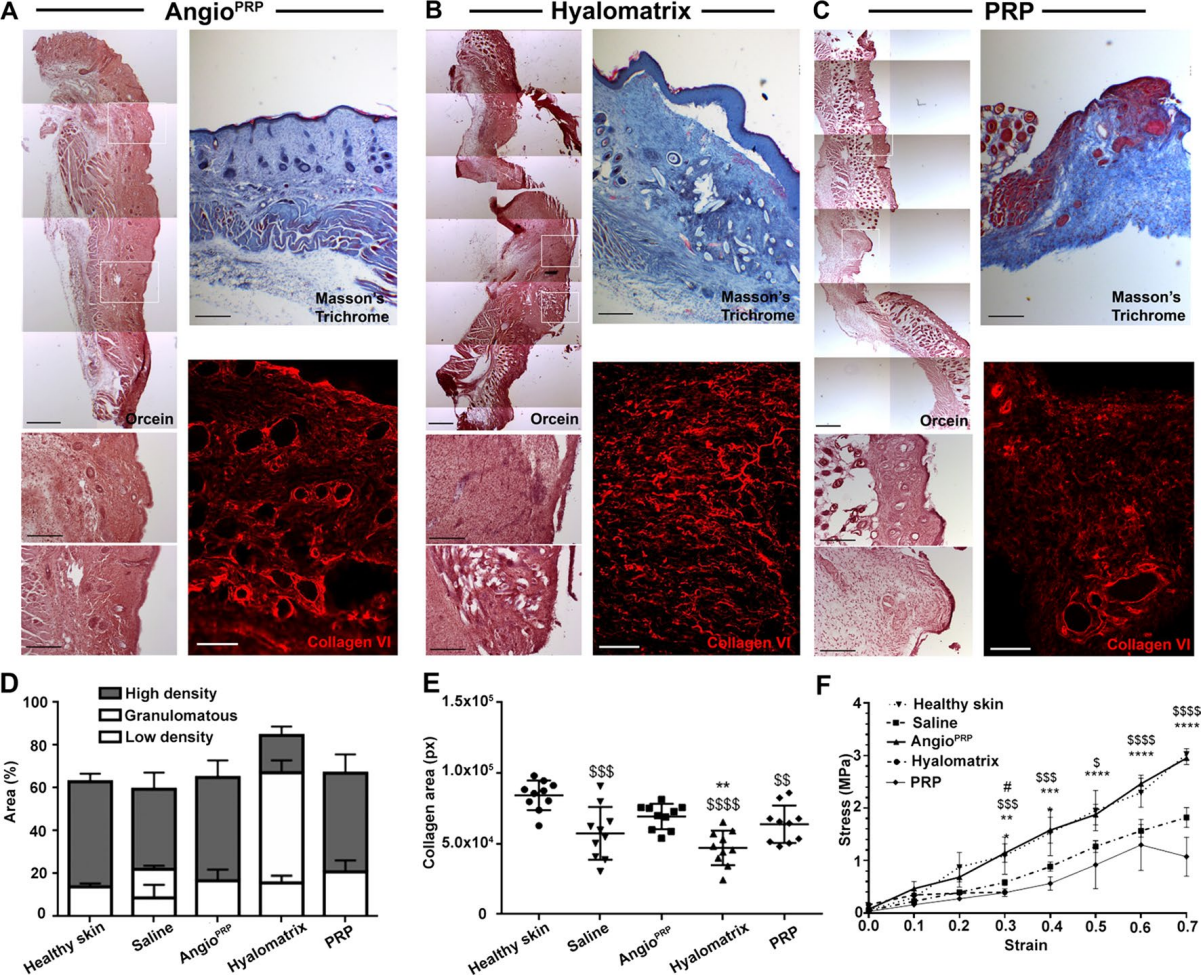
Skin elastic properties. Representative images of orcein histological staining of skin tissues of the NOD.Cg-Prkdc^Scid^/J mice treated with Angio^PRP^ (**A**), Hyalomatrix (**B**) and PRP (**C**) 21 days after wounding (scale bar = 500 µm). Frame magnifications are shown in the enlarged images (scale bar = 200 µm); Masson’s trichrome (scale bar = 500 µm) and collagen VI (scale bar = 100 µm) immunofluorescence stainings are shown in right panels of **A**, **B** and **C**. **D** Orcein staining quantification of high and low-density elastin and granulomatous area in skin tissues 21 day after treatment. Collagen VI area quantification is reported in **E** (one-way ANOVA analysis of variance with Bonferroni correction; ^$^comparison to healthy skin, *comparison to Angio^PRP^, **p < 0.01 ***p < 0.001 ****p < 0.0001). **F** Graphical representation of stress–strain curves obtained from dorsal skin analysis to determine mechanical properties of skin treated with Angio^PRP^, Hyalomatrix and PRP compared to saline and healthy skin (multiple *t* test; *Angio^PRP^ vs Hyalomatrix; ^$^Angio^PRP^ vs saline; ^#^Angio^PRP^ vs PRP; *p < 0.05 **p < 0.01 ***p < 0.001****p < 0.0001)

We examined the beneficial effects on the healing of the skin wounds of Angio^PRP^ (*n* = 10), hyalomatrix (*n* = 10) and PRP (*n* = 10) that were placed below full-thickness excisional wounds (diameter, 5 mm) on mouse’s dorsal skin (NOD.Cg-Prkdc^Scid^/J mice as animal model for human products engraftment (Fig. 4A). All treatments were performed at day of wounding. The control group received saline solution. In all groups, we found surrounding epithelium forming an epithelial tongue as the first layer advanced toward the wound [41]. On day 7, the Angio^PRP^ group presented the fastest coverage of the wound compared to PRP group (65.35% and 51.34% respectively, *p* < 0,0001), whereas the outer silicone barrier layer of Hyalomatrix [42] seemed to impede the advancement of the surrounding epithelium (Fig. 4B). The wound healing rate was substantially accelerated by Angio^PRP^ (Fig. 4A, B). By day 21, Angio^PRP^ induced a complete wound closure, whereas hyalomatrix and saline solution achieved 75–80% and 90% of wound closure (*p* < 0.0001) (Fig. 4B). Delayed wound healing of saline solution and hyalomatrix treatments was also confirmed by the quantification of time needed for the achievement of 60% of wound closure (mean of 10.5 and 13 DPI respectively for saline solution and hyalomatrix vs 6.5/7 DPI for Angio^PRP^). On day 21, re-epithelialization was complete in Angio^PRP^ group that showed mature stratified epithelia (Fig. 4C). Immunofluorescent staining and quantification of cytokeratin 5 (keratin secreted by keratinocytes in basal layer) and cytokeratin 10 (keratin secreted by differentiated keratinocytes in suprabasal layers) showed a more intense fluorescence for Angio^PRP^ compared to hyalomatrix (7.24 ± 2.97 × 10^5^ a.u. and 1.93 ± 1.69 × 10^5^ a.u. respectively for cytokeratin 5, p < 0.0001; 5.30 ± 1.72 × 10^5^ a.u and 2.55 ± 1.56 × 10^5^ a.u. respectively for cytokeratin 10, *p* = 0.0124), PRP (3.03 ± 0.92 × 10^5^ a.u. for cytokeratin 5, *p* < 0.0001) and saline solution (2.79 ± 1.69 × 10^5^ a.u. for cytokeratin 5, *p* < 0.0001) (Fig. 4D, E and Supplementary Fig. S2). On day 21, the intact dorsal NOD.Cg-Prkdc^Scid^/J healthy skin (Supplementary Fig. S2A) and Angio^PRP^ wounds (Fig. 4D, E) revealed similar formation of stratified epithelia. Kinetic analysis of Angio^PRP^ wound closure demonstrated granulation tissue filled in the dermis and proliferation of the wound edge keratinocytes on day 14 with subsequent complete closure on day 21 (Supplementary Fig. S3A), confirmed by the presence of E-cadherin-positive adherent junction throughout the original lesion (Supplementary Fig. S3B). Immunofluorescence analysis for human-specific human nuclei confirmed the presence of Angio^PRP^ cells in the wound space on day 7 and in stratified epithelia on day 21 (Fig. 4F). Angio^PRP^ group had more regenerated hair follicles and sebaceous glands than other treatment groups (Fig. 4C). Our next target was to verify the extracellular matrix remodeling and the physical properties of treated wounds. Histochemical staining with orcein demonstrated an elastin composition of Angio^PRP^-treated wounds more similar to healthy skin than hyalomatrix and PRP-treated tissues (Figs. 5A–C, S3). Moreover, hyalomatrix and saline solution-treated skin samples showed the presence of a granulomatous buildup of cells filling the wound bed (Figs. 5B, D, S3B). Additionally, Masson’s trichrome staining revealed biomaterial fibers entrapment inside the lesion area of hyalomatrix-treated wound that might impede the advancement of the surrounding epithelium (Figs. 5B, S4). As collagen and elastin control the elasticity of the connective [43], we examined the expression pattern of type VI collagen in healthy skin, Angio^PRP^, hyalomatrix, PRP and saline-injected wounds. The immunofluorescence staining revealed that type VI collagen was more present in Angio^PRP^ wounds (6.92 ± 0.89 × 10^4^ pixels.) and healthy skin (8.43 ± 1.04 × 10^4^ pixels) than hyalomatrix (4.71 ± 1.22 × 10^4^ pixels), PRP (6.38 ± 1.32 × 10^4^ pixels) and saline-injected wounds (5.73 ± 1.86 × 10^4^ pixels) (Fig. 5E and Supplementary Fig. S3). We next examined the mechanical properties of healthy skin compared to the Angio^PRP^, hyalomatrix, PRP and saline-injected wounds. During mechanical testing, resistance to tension by fibrils results in the linear region of the stress–strain curve, the modulus of which is often defined as modulus of elasticity. Angio^PRP^ wounds showed similar modulus as compared to healthy skin (3.2 MPa ± 0.1 and 3.1 Mpa ± 0.1 respectively). In contrast, a lower modulus of elasticity was found in hyalomatrix, PRP and saline-injected wounds (0.38 Mpa ± 0.03, 1.9 Mpa ± 0.4 and 1.8 Mpa ± 0.4, respectively) (Fig. 5F). Moreover, stress–strain curves of tissues treated with Angio^PRP^ were significantly higher than PRP (*p* < 0.0001), hyalomatrix (*p* = 0.08) and saline solution (*p* < 0.0001) (Fig. 5F). These changes are consistent with our observation of the delayed matrix remodeling in hyalomatrix and saline-injected wounds. Therefore, Angio^PRP^ promoted faster wound healing and increased regeneration of cutaneous appendages compared to other treatments.

### Angio^PRP^ reduced inflammation and promoted angiogenesis throughout the repair of skin mouse wounds

**Fig. 6:**
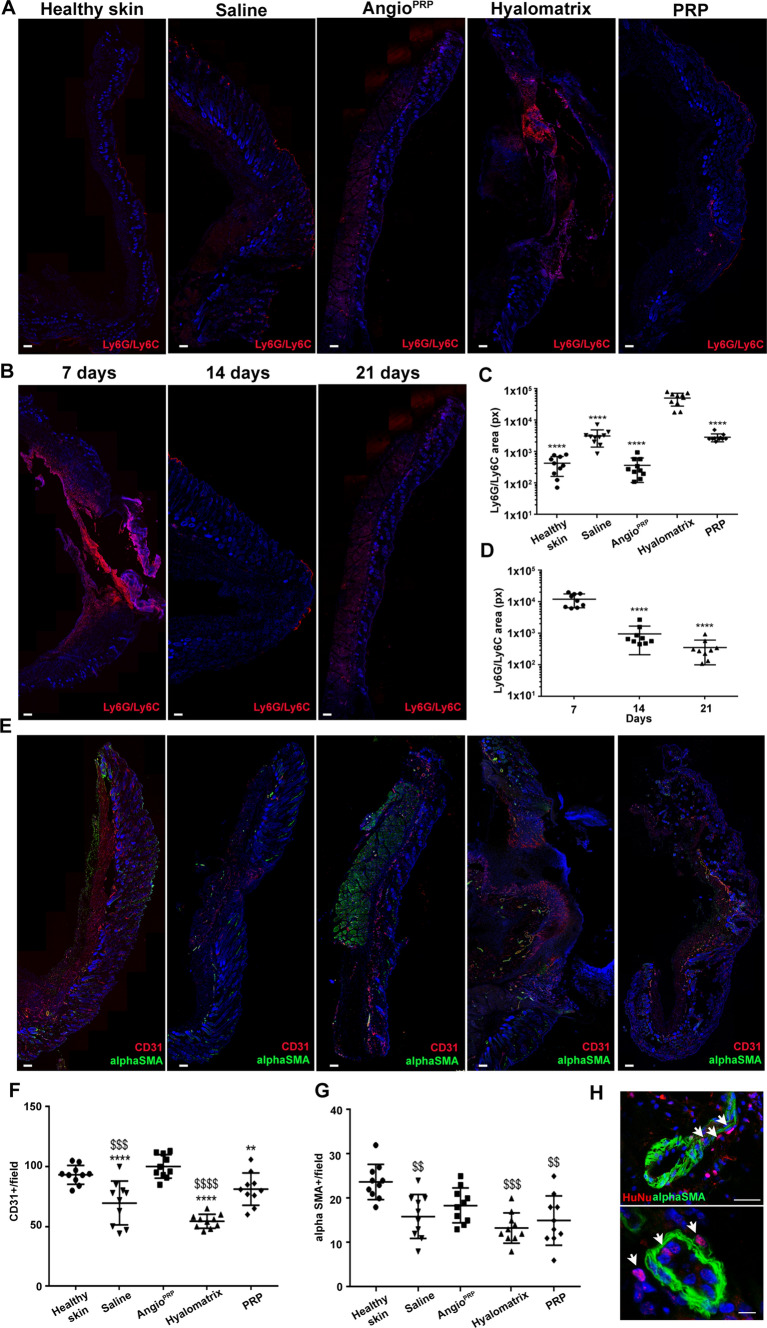
Inflammatory and vascularization processes. **A** Overview images of Ly6G/Ly6C immuno-fluorescence staining of healthy skin, saline, Angio^PRP^, Hyalomatrix and PRP-treated wounds at 21 DPI (scale bar = 250 µm). **B** Overview images of Angio^PRP^-treated wounds displaying Ly6G/Ly6C at 7, 14, 21 DPI (scale bar = 250 µm). **C** Fluorescence quantification of Ly6G/Ly6C at 21 DPI for all conditions tested (one-way ANOVA analysis of variance with Bonferroni correction; *comparison to hyalomatrix; ****p < 0.0001). **D** Fluorescence quantification of Ly6G/Ly6C at 7, 14, 21 days after Angio^PRP^ treatment (one-way ANOVA analysis of variance with Bonferroni correction; *comparison to 21 days; ****p < 0.0001). Data are presented as mean ± SD. **E** Overview images of immunofluorescence staining for CD31 (red) and α-SMA (green) in all tested conditions at 21 DPI (scale bar = 250 µm). Quantification of CD31 (**F**) and α-SMA (**G**)-positive cells per field in healthy skin, saline, Angio^PRP^, hyalomatrix and PRP-treated skin 21 days after injury (one-way ANOVA analysis of variance with Bonferroni correction; ^$^comparison to healthy skin, *comparison to Angio^PRP^; **p < 0.01 ***p < 0.001 ****p < 0.0001). **H** Immunofluorescence staining for α-SMA and human nuclei (HuNu) (arrows) after 21 days of Angio^PRP^ treatment (scale bar = 25 µm for top panel and scale bar = 10 µm for lower panel)

To evaluate differential immune host response elicited by each treatment on day 21, we performed immunofluorescence staining for neutrophils and monocyte/macrophages with anti-Ly6G/Ly6C antibody. The Angio^PRP^ group revealed a significant decrease of Ly6G/Ly6C+ cells compared to the hyalomatrix (*p* < 0.0001) and saline solution-treated wounds (Fig. 6A, C) suggesting more substantial immune infiltration. Likewise, the kinetic of Ly6G/Ly6C in Angio^PRP^-treated wounds displayed a progressive reduction during the weeks after treatment (1.23 ± 0.57 × 10^4^ a.u at 7 DPI; 9.69 ± 7.57 × 10^2^ a.u. at 14 DPI and 3.61 ± 2.60 × 10^2^ a.u. at 21 DPI; *p* < 0.0001) (Fig. 6B–D).

To explore whether treatments stimulated angiogenesis, we characterized and quantified the number of CD31-positive capillaries and α-SMA-positive vessel structures in all treated wounds on day 21. Interestingly, Angio^PRP^ and healthy skin displayed similar number of CD31 and α-SMA-positive vessels (100.2 ± 9.8 and 93.3 ± 7.8 respectively for CD31; 18.4 ± 3.95 and 23.7 ± 3.97 respectively for α-SMA) (Fig. 6E–G). Otherwise, hyalomatrix, PRP and saline solution-treated wounds showed a reduced number of CD31 per field compared to Angio^PRP^-treated wounds (54.4 ± 5.8 *p* < 0.0001; 81.2 ± 13.46, *p* = 0.0085; 69.7 ± 18.29 *p* = 0.0006 respectively), while α-SMA-positive vessels were significantly decreased only compared to healthy skin (13.3 ± 3.4 *p* < 0.0001; 15 ± 5.57, *p* = 0.0007; 15.9 ± 4.95, *p* = 0.0029 respectively) (Fig. 6F, G). Of note, human nuclei + cells were found located around α-SMA + vessels in Angio^PRP^-treated wounds on day 21 (Fig. 6H). In summary, Angio^PRP^ led to reduced inflammation and enhanced angiogenesis.

### Protein profile of Angio^PRP^ wound healing

**Fig. 7:**
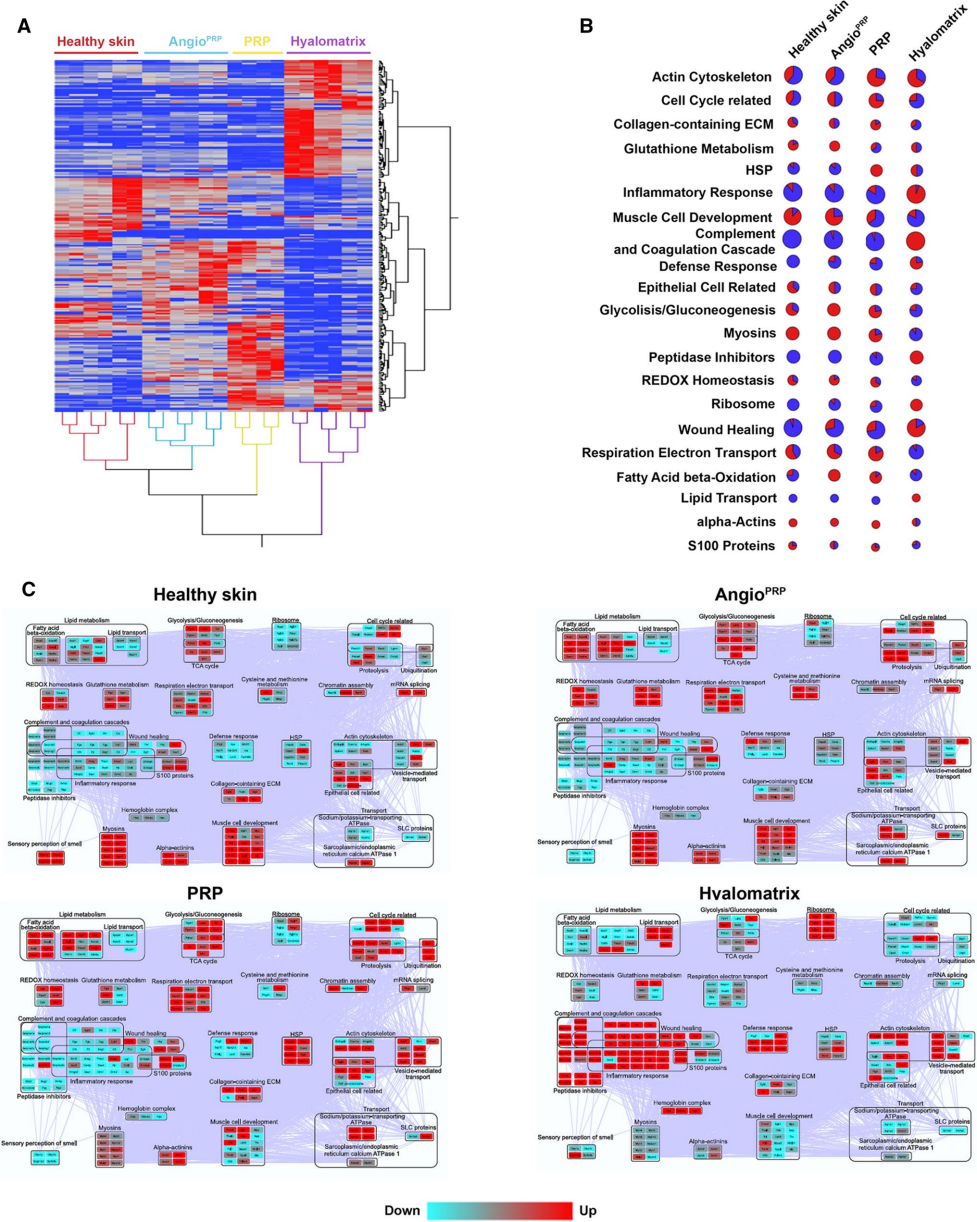
Molecular pathways involved in Angio^PRP^ wound healing. **A** Hierarchical clustering of proteins differentially expressed (DEPs, *n* = 254, *p* < 0.01) by comparing healthy *vs* Angio^PRP^, PRP and hyalomatrix-treated skin samples 21 days after injury. Clustering was performed by computing the average spectral count (aSpC) value of proteins selected by linear discriminant analysis (LDA); *Euclidean*’s distance metric and *Ward*’s method were applied. The heat map is related to the normalized aSpC (range 0–100) and indicates down- (blue) and up-regulated (red) proteins, respectively. Each sample has been tested with two technical replicates. **B** Enrichment of main functional categories of low (blue) and high (red) abundant proteins in healthy skin, Angio^PRP^, PRP and hyalomatrix; size of bubbles is indicative of the number of proteins involved in each pathway. **C** Protein–protein interaction (PPI) network (254 nodes and 6611 edges) reconstructed starting from DEPs selected by comparing healthy vs Angio^PRP^, PRP and Hyalomatrix treated skin. The network was reconstructed by StringApp and considering exclusively physical and functional PPIs. Based on GO terms, DEPs were grouped in 33 distinct functional modules. Red color code indicates up-regulated proteins, while blue color code indicates those down-regulated

To ascertain the recovery performance of wound healing treatments, we performed proteomic assay for the Angio^PRP^, hyalomatrix and PRP samples harvest on day 21 and compared them to healthy dorsal skin of NOD.Cg-Prkdc^Scid^/J mice. Globally, 2268 distinct proteins were identified following a total number of 22 LC/MS runs (Supplementary Table 1). This data matrix, processed by LDA, allowed the selection of 254 differentially expressed proteins (DEPs); specifically, 236 DEPs in hyalomatrix samples (110 up-regulated and 126 down-regulated), 123 DEPs in PRP (85 up-regulated and 38 down-regulated) and 185 DEPs in Angio^PRP^ (92 up-regulated and 93 down-regulated), while 157 DEPs were found by comparing Angio^PRP^ vs PRP (93 up-regulated in Angio^PRP^ (down-regulated in PRP) and 63 up-regulated in PRP (down-regulated in Angio^PRP^) (Supplementary Table 2). The higher similarity between Angio^PRP^ and healthy proteome was further confirmed by hierarchical clustering (Fig. 7A); healthy skin and Angio^PRP^ overlapped in the same macro group, distinct from the PRP and hyalomatrix that behave in different clusters (Fig. 7A). However, although less than Angio^PRP^, in comparison to hyalomatrix treatment also PRP induced a proteome recovery toward a healthy state. In fact, following treatment with Angio^PRP^ or PRP, proteins involved in inflammatory response, complement and coagulation cascades, peptidase inhibitors, S100 proteins and wound healing were restored to levels similar to healthy (Fig. 7B, C), while they were still activated after hyalomatrix treatment. In addition, Angio^PRP^ and PRP treatments induced up-regulation of functional modules involved in fatty acid beta oxidation and lipid metabolism, REDOX homeostasis, glutathione metabolism, amino acid metabolism, mitochondrial respiration, myosin, muscle cell development, cell cycle and proteolysis. On the contrary, ribosomes, actin cytoskeleton, vesicle-mediated transport, lipid transport, hemoglobin complex and defense response modules were specifically activated by hyalomatrix treatment. Of note, some proteins involved in sensory perception of smell resulted up-regulated in healthy and down-regulated in all Angio^PRR^, PRP and hyalomatrix samples (Figs. 7C). Moreover, cellular stress HSP resulted to be up-regulated in PRP condition compared to Angio^PRP^ (Fig. 7B, C). Inside functional modules affected by the considered therapeutic treatments, we identified a wound healing fingerprint of 30 proteins for Angio^PRP^ including GSTZ1 and GSTT3 involved in glutathione metabolism, PHGDH, PRG2 involved in defense response and cell cycle-related TUBAL3 (Supplementary File 2). Compared to PRP-treated wound, Angio^PRP^ samples displayed an up-regulation of proteins involved in muscle cell development, such as CFL2 and FHL1 (Supplementary File 2) and myosins (Fig. 7B); whereas, peptidase inhibitors, such as SERPINA3N, SERPINA3M, SERPINB1A, displayed similar values in healthy skin and Angio^PRP^ samples (Supplementary File 2). Complement and coagulation cascade factors, as C3 and CFH, resulted up-regulated only in hyalomatrix-treated samples, while in Angio^PRP^- and PRP-treated tissues their expression is comparable to healthy skin (Fig. 7B, C). Overall, these results indicate a more efficient system of detoxification and REDOX metabolism in Angio^PRP^-treated wounds, with a more functional recovery of skin in terms of mechanical and structural properties.

## Discussion

The physiological healing of wounds is governed by highly effective sequence of events that can be restricted by the extension of the affected area as well as by patient-related factors that include nutritional status, diabetes, pre-existing skin disease, such psoriasis and genetic susceptibility [43, 44]. The skin-wound repair process can be divided into four phases: hemostasis (hours), inflammation (days), proliferation (1 to 2 weeks), and remodeling (> 2 weeks). The influence of the immune microenvironment on tissue generation is in part determined by a variety of signals released from immune cells that modulated the behaviors of keratinocytes and fibroblasts. Conversely, keratinocytes and fibroblasts secreted chemotactic pro- or anti-inflammatory signals that regulated immune cell polarization and function. However, the communication network of immune and cutaneous cells is complicated, and wound healing resulted from the combined effect of these factors. Different approaches are currently used, with a focus on the autologous products that offer a direct source of growth factors from patient’s blood. Nowadays, the wound management is impacted by excessive costs and detrimental physical and psychological side effects for patients. Here, we developed a highly efficient high-throughput protocol based on a single-use sterile closed system (Sep4Angio™) to collect a human blood-derived product named Angio^PRP^ that is mainly composed by platelets and distinct proportion of angiogenic Tangs and EPCs. Functionally, the Angio^PRP^ promotes angiogenesis of HUVEC endothelial cells and proliferation of fibroblasts and keratinocytes, contributing to accelerate the wound healing of damaged organotypic human skin (Figs. 2, 3). Moreover, the Angio^PRP^ organotypic wounds revealed the correct maturation of the spinous and cornified layers. To explore whether the Angio^PRP^ had a similar performance in vivo, we used it in immuno-deficient mouse skin-wound model. We demonstrated that Angio^PRP^ led to faster wound healing and enhanced regeneration of the basal and granular layers and vessel remodeling of cutaneous wounds compared to other treatments, such as PRP or hyalumatrix. Hair follicle regeneration was also observed in Angio^PRP^ wounds. Correspondingly, Angio^PRP^ promoted the decrease in monocyte/macrophages infiltrates which is essential for wound remodeling and healing and to avoid chronic inflammation [45]. Those effects were coupled with normalization of mechanical properties of Angio^PRP^ wounds which is sustained by a correct arrangement of elastin and collagen fibers (Fig. 5). Further, proteomic assay for the Angio^PRP^ and control healthy samples revealed comparable levels of proteins involved in epithelization, muscle development and cytoskeleton stabilization (Supplementary File 2). Meanwhile, we identified a specific Angio^PRP^ metabolic signature enriched in proteins related to glutathione metabolism and therefore redox pathway. Limitations of this study include the insufficient disclosure of how Angio^PRP^ cells participate in vessel neogenesis and their long-term distribution was not fully disclosed. However, our biological and mechanical findings are potentially applicable in the design of further clinical trial in wound repair, thereby improving patient outcomes.

## Conclusion

Over the past years, growth factors mediated by PRP and cell-based therapies were developed to improve wound healing. Unfortunately, clinical trials of single PRP or cell replacement treatments resulted in poor outcomes. Instead, a combined treatment composed of PRP and a pool of pro-angiogenic/keratogenic cells may provide a more integrated method for a therapeutic approach to actively improve wound healing. The results of our study highlight the power of Angio^PRP^ treatment to enhance wound healing by promoting a cascade of events leading to the inflammatory reduction, re-epithelialization and blood vessel regeneration. Taken together, we demonstrate that Angio^PRP^ retains a regenerative capacity by improving the wound repair and we provide insights into the Angio^PRP^ molecular mechanism opening new perspectives in the treatment of skin injuries.

## Materials and methods

### Angio^PRP^ isolation and characterization

We designed a sterile and closed class IIa device (Sep4Angio™), characterized by a collecting tube with an inert porous membrane of high-grade polyethylene, a rubber stopper to insert peripheral blood with a 2.5 ml syringe needle (21G) and a ring nut to adjust the plasma phase volume above the membrane after centrifugation (Fig. 1A, B). The device was designed for single use only and to collect blood-derived mononucleated cells and the plasma phase after centrifugation without opening the system. Peripheral blood was collected from healthy volunteers (*n* = 101) of the blood bank of Department of Transfusion Medicine and Haematology at Policlinico Hospital of Milan, after informed consent and according to the guidelines approved by the Ethics Committee on the Use of Human Subjects in Research of the Policlinico Hospital of Milan (Milan, Italy, Ethics Committee permission number 793/13). 2.5 ml of peripheral blood, collected in sodium citrate tube were filled into Sep4Angio™ device and centrifuged at 460*g* for 5 min to induce the phase separation (EP20161201.7). The platelet-rich-plasma phase (PRP) and the cells at the interface between red cells and plasma were collected. We analysed pre-separation blood and Angio^PRP^ by blood Coulter counter instrument (Sysmex XN-1000). Pre-separation blood and cell phase collected were directly labelled with monoclonal antibodies shown below. Cells were incubated with Syto 16, anti-CD45 V500, anti-CD3 V450, anti-CD3 APC, anti-CD56 PE-CY7, anti-CD14 APC-H7, anti-CD16 PE, anti-CD15 V450, anti-CD19 APC-R700 or anti-CD31 PE Cy7, anti-CD184 APC, anti-CD90 PerCP, anti- CD90 FITC, anti-CD146 PE, anti CD34 APC (BD Biosciences-Pharmingen, San Diego, California, USA). The controls were isotype-matched mouse immunoglobulins. After each incubation performed at 4 °C for 20 min, cells were washed in saline solution 1X containing 1% heat-inactivated FCS and 0.1% sodium azide. The cytometric analyses were performed on a LYRIC flow cytometer using FACSuite software (BD Biosciences-Immunocytometry System). Each analysis included at least 1–2 × 10^4^ events for each gate. A light-scatter gate was set up to eliminate cell debris from the analysis. The percentage of positive cells was assessed after correction for the percentage reactive to an isotype control conjugated to a specific fluorophore. Percentage of different cells subpopulations was calculated on the Syto 16-positive gate.

### Ex vivo preclinical experimentation: EPC colony-forming assay, HUVEC co-culture and organotypic skin culture

The angiogenic potential of Angio^PRP^ was tested in 35-mm dishes using the Endothelial Progenitor Cell Colony-Forming Assay (EPC-CFA) (MethoCult SFBIT; STEMCELL Technologies Inc.) added with proangiogenic growth factors/cytokines, as previously reported [40] (rh SCF 100 ng/ml, rh VEGF 50 ng/ml, rh b-FGF 50 ng/ml, rh EGF 50 ng/ml and rh IGF-1 ng/ml, all from Miltenyi Biotec; heparin 2U/ml, STEMCELL Technologies Inc). Aliquots of Angio^PRP^ were seeded at a cell density of 5 × 10^4^ cells/dish (3 dishes per volunteer). 16 to 18 days after the beginning of the culture, the number of adherent EPC colonies per dish was counted under phase contrast light microscopy LEICA DMi8 (Leica, Germany). Primitive EPC colony-forming units (pEPC-CFUs) and definitive EPC-CFUs (dEPC-CFUs) were separately counted and expressed as a percentage of the total number. Pro-angiogenic potential of Angio^PRP^ was evaluated in co-culture system constructed using human umbilical vein endothelial cells (HUVECs) as previously described [46]. Briefly, 8 × 10^4^ HUVEC (ATCC-LGC, VA, USA) were plated on 3D Matrigel (BD Biosciences-Pharmingen, San Diego, California, USA) and co-cultured with different cell products, as described below. For GFP Angio^PRP^, the entire product obtained from Sep4Angio™ device separation (7.3 × 10^5^ cells) was infected with GFP vector and then co-cultured with HUVEC cells. For Angio^PRP^-Tang^GFP^, cell product obtained from Sep4Angio™ separation (7.7 × 10^5^cells) was sorted for Syto16+/CD45+/CD3+/CD31+/CD184+ population (Tang; gate strategy shown in Supplementary Fig. S1B); cells obtained (1.8 × 10^5^ cells) were infected with GFP vector and collected together with negative fraction. For Angio^PRP^-EPC^GFP^ cell product obtained from four Sep4Angio™ devices, separation (3 × 10^6^ cells) was sorted for Syto16+/CD45+/CD31+/CD90+/CD146+ population (EPC; gate strategy shown in Supplementary Fig. S1B); cells obtained (1.2 × 10^4^ cells) were infected with GFP vector and collected together with negative fraction. 10^8^ ip/ml were used to transduce EPC and Tang cells using lentiviral vector: pLENTI-CAG (GFP)-Rsv (puro). 1.2 × 10^4^ EPC and 1.8 × 10^5^ Tang cells were plated in 48-well tissue culture dishes coated with fibronectin. Cells were infected in 250 µl of RPMI supplemented with cytokines [47] and incubated for 24 h at 37 °C and 5% CO_2_. 24 h post transduction, supernatant was discarded and, after washing with saline solution 1×, cells were collected together with negative fraction and PRP and used for co-cultured experiments. For Angio^PRP^-Tang^NEG^ and Angio^PRP^-EPC^NEG^, the negative fractions were suspended in PRP and added to HUVEC for co-culture experiments. For all the conditions, the equivalent of 50 µl of single product was tested in co-culture with 8 × 10^4^ HUVEC; after 24 h, cells’ ramification was quantified as number of nodes, number of segments and total mesh area per field using ImageJ software (Angiogenesis analyze, NIH) [48]. To investigate the skin regeneration potential of Angio^PRP^, we used a multilayered model of human dermis and epidermis as previously described (MatTek’s EpiDermFT Full Thickness EFT-400) [49]. Epidermal-only wounds were induced using a sterile 5 mm dermal biopsy punch (Miltex Inc., York, PA) and the epidermis was mechanically removed using forceps. After wounding, EpiDermFT tissues were cultured into 6-well plate with four different culture conditions: (1) an organotypic skin culture with 3.5 × 10^4^ cells and 9.85 × 10^6^ platelets for complete Angio^PRP^, (2) 3.5 × 10^4^ cells of Angio^cells^ suspended in saline solution, (3) 9.85 × 10^6^ platelets for PRP, (4) 50 µl of saline solution 1× (as negative control) and analyzed after 24 h, 2, 4, 5, 6 and 7 days of culture. Blood from 10 healthy volunteers was collected in Sep4Angio™ device to obtain 10 individuals Angio^PRP^ as described above. The isolated 10 Angio^PRP^ were pooled and further centrifuged at 1500 rpm for 10 min to obtain a pellet of cells which were suspended in saline solution (Angio^cells^). The supernatant containing platelet-rich plasma (PRP) was used as such. Wound closure was calculated via equation:

$$\begin{aligned}&{\text{Wound }}\,{\text{healing }}\left( \% \right)\\& \quad = \, \left( {{1} - {\text{Open}}\,{\text{wound}}\,{\text{area}}/{\text{Initial}}\,{\text{wound}}\,{\text{area}}} \right) \, \times 100. \end{aligned}$$

### In vivo wound healing experiments

Five-month-old severe combined immuno-deficient (NOD.Cg-Prkdc^Scid^/J) [50] mice were obtained from Charles River Laboratories International, Inc. (Calco, Italy); the use of animals in this study was authorized by the National Ministry of Health (authorization number 51/2018-PR). All experimental protocols were reviewed and approved by the University of Torino’s animal ethics research committee. The methods described below were carried out in accordance with those approved protocols, as well as the Italians ethical guidelines regarding the use of experimental animals. *N* = 10 animals per treatment were used for the experimental section at 21 days (Angio^PRP^, hyalomatrix and PRP; saline solution was included in each animal as control); for the experimental session of Angio^PRP^ analysis at 7, 14 and 21 days after injury, *n* = 3 animals were used for each time point. Wound healing model was obtained as described in Dunn and colleagues [51]. Briefly, animals were anesthetized with avertin and two full-thickness excisions of 5 mm that include the panniculus carnosus were created on the dorsum, one on each side of the midline of the mouse. A silicone splint was placed around the wound with the assistance of adhesive and the splint was then secured with interrupted sutures. Each mouse acts as its own control, with one wound receiving treatment (Angio^PRP^, PRP or Hyalomatrix, Anika Therapeutics Inc., Bedford, MA 01730, USA) and the other phosphate-buffered saline (saline solution 1×). A transparent occlusive dressing was applied to prevent contamination. Wounds were checked by taking photos every 2–3 days, and the area was quantified relative to a millimeter reference using ImageJ software (NIH) and expressed as the percentage of wound area measured at day 0, 4, 7, 10, 14 and 21 days after injury, corresponding to wound closure; mice were sacrificed by cervical dislocation under full deep anesthesia and the back skin lesions were removed; the biopsies have been divided into two group respectively for histological or proteomic analysis. One group was placed in iso-pentane and froze at − 80 °C for proteomic analysis. The other group was incubated in 4% paraformaldehyde in saline solution at 4 °C overnight and after transferred to 30% sucrose in saline solution 1× solution for a further 24 h at 4 °C, embedded in O.C.T matrix and froze at − 80 °C. Serial sections of 12 μm thickness were cut and examined by immunofluorescence and histological analysis.

### Histological and immunofluorescence staining

Serial sections of 12 μm of skin tissue and organotypic skin were cut and stained with hematoxylin and eosin (H&E, Bio Optica Spa, Italy) Orcein (Sigma-Aldrich Inc., St. Louis, MO, USA) and Masson’s trichrome staining (Bio Optica Spa, Italy), according to the manufacturer’s instructions for morphological assessment. Images were captured with LMD6000B (Leica, Germany) at 12 regular intervals, representing the entire section and the epidermal thickness was quantified as area per interval using ImageJ software (http://rsbweb.nih.gov/ij/). For immunofluorescence analysis, transversal tissue sections were incubated with mouse monoclonal antibody anti-cytokeratin 10 (1:100, ab9025, Abcam, UK), rabbit monoclonal antibody anti-vimentin (1:100, ab16700, Abcam, UK), rabbit polyclonal antibody anti-involucrin (1:100, ab53112,Abcam, UK), mouse monoclonal antibody anti-cytokeratin 14 (1:100, ab7800 Abcam, UK) rabbit polyclonal antibody anti-cytokeratin 5 (1:100, ab53121, Abcam, UK), rabbit polyclonal antibody anti-β-catenin (1:200, ab16051, Abcam, UK), Alexa Fluor 594 rat monoclonal antibody anti-Ly-6G/Ly-6C (Gr-1)(1:50, 108448 BioLegend), rat monoclonal antibody anti-CD31 (1:50, 550274 BD Biosciences-Pharmingen, San Diego, California, USA), rabbit polyclonal antibody anti-collagen VI (1:250, ab6588, Abcam, UK), mouse monoclonal antibody anti-alpha SMA (1:50, A2547, Sigma-Aldrich Inc., St. Louis, MO, USA), rabbit polyclonal antibody anti-VE-cadherin (1:50, ab33168, Abcam, UK), mouse monoclonal antibody anti-eNOS (1:100, ab76198, Abcam, UK), rat monoclonal anti-E-cadherin (1:100, ab11512, Abcam, UK), mouse monoclonal anti-cytokeratin 10 (1:100, ab9025, Abcam, UK), rabbit polyclonal anti-loricrin (1:100, ab85679, Abcam, UK) and mouse monoclonal antibody anti-human nuclei (1:100, MAB1281, Chemicon, California, USA). Cell nuclei were stained for 5 min at room temperature with DAPI (Sigma-Aldrich Inc., St. Louis, MO, USA). Slides were analyzed using a fluorescent microscope LEICA DMi8 (Leica, Germany), images were captured at regular intervals along the entire section and fluorescence intensity per single interval was quantified with Image J software (http://rsbweb.nih.gov/ij/); integrated density was measured using a ROI corresponding to epidermal region in each slice interval and plotted in the graphic after subtracting the corresponding background signal measured within the tissue-free area [52].

### Strength measurements

Following sacrifice, the skins for mechanical testing were placed in metal screw clamps with rubber pieces covering the clamped ends. Clamps were placed into a Bose Electroforce 3100 instrument. Applying an initial traction of 0.15 N, the traction measured in MPa was increased by 0.2% per second up to the breaking point. Force (*N*) and displacement (mm) were measured on a *xy* plotter and these points were subsequently recorded as stress (*σ* = force per cross-sectional area) and strain (*ε* = change in length/initial length) and re-plotted in Excel [53].

### Proteomics analysis

#### In-solution digestion

For proteomic analysis, the epidermal and dermal layers of the treated skin enclosed by the silicone splint were removed 21 days after injury and frozen in iso-pentane. Samples were then suspended in 200 µl 0.1 M NH_4_HCO_3_ pH 7.9 buffer and homogenized in ice. The protein concentration was assayed using SPN-Protein assay kit (G-Biosciences, St. Louis, MO, USA) and the membrane proteins were solubilized by adding Rapigest SF reagent (Waters Co, Milford, MA, USA) at the final concentration of 0.2% (w/v). The resulting suspensions were incubated under stirring at 100 °C for 20 min and at 80 °C for 2 h. The digestion was carried out on 50 ± 0.5 µg proteins of each sample by adding Sequencing Grade Modified Trypsin (Promega Inc., Madison, WI, USA) at an enzyme/substrate ratio of 1:50 (w/w) overnight at 37 °C in 0.1 M NH_4_HCO_3_ pH 7.9 buffer with 10% CH_3_CN. An additional aliquot of 0.5 µg of trypsin (1:100 w/w) was added in the morning, and the digestion continued for 4 h. Moreover, the addition of 0.5% tri-fluoro-acetic acid (TFA) (Sigma-Aldrich Inc., St Louis, MO, USA) stopped the enzymatic reaction, and a subsequent incubation at 37 °C for 45 min completed the RapiGest acid hydrolysis [54]. The water immiscible degradation products were removed by centrifugation at 13,000 rpm for 10 min. Finally, the tryptic digest mixtures were desalted using Pierce C-18 spin columns (Thermo Fisher Scientific—Pierce Biotechnology, Rockford, Il, USA), according to manufacturer protocol and were re-suspended in 0.1% formic acid (Sigma-Aldrich Inc., St. Louis, MO, USA) in water (LC–MS Ultra CHROMASOLV, Honeywell Riedel-de Haen, Muskegon, MI, USA).

#### LC–MS/MS

Proteomics analyses by LC–MS were performed as previously described [55]. Briefly, trypsin digested mixtures were analyzed using Eksigent nanoLC-Ultra 2D System (Eksigent, part of AB SCIEX Dublin, CA, USA) combined with cHiPLC-nanoflex system (Eksigent) in trap-elute mode. Briefly, samples (0.8 µg injected) were first loaded on the cHiPLC trap (200 µm × 500 µm ChromXP C18-CL, 3 µm, 120 Å) and washed with the loading pump running in isocratic mode with 0.1% formic acid in water for 10 min at a flow of 3 µl/min. The automatic switching of cHiPLC ten-port valve then eluted the trapped mixture on a nano-cHiPLC column (75 µm × 15 cm ChromXP C18-CL, 3 µm, 120 Å) through an 87 min gradient of eluent B (eluent A, 0.1% formic acid in water; eluent B, 0.1% formic acid in acetonitrile) at a flow rate of 300 nl/min. In depth, gradient was: from 5 to 10% B in 3 min, 10 to 40% B in 80 min, 40 to 95% B in 17 min and holding at 95% B for 7 min. Trap and column were maintained at 35 °C for retention time stability. Mass spectra were acquired using a QExactive mass spectrometer (Thermo Fisher Scientific, San Josè, CA, USA), equipped with an EASY-Spray ion source (Thermo Fisher Scientific, San Josè, CA, USA). Easy spray was achieved using an EASY-Spray Emitter (Dionex Benelux BV, Amsterdam, The Netherlands) (nanoflow 7 µm ID Transfer Line 20 µm × 50 cm) held to 1.9 kV, while the ion transfer capillary was held at 220 °C. Full mass spectra were recorded in positive ion mode over a 400–1600 *m/z* range and with a resolution setting of 70,000 FWHM (@ *m/z* 200) with 1 micro-scan per second. Each full scan was followed by 10 MS/MS events, acquired at a resolution of 17,500 FWHM, sequentially generated in a data-dependent manner on the top ten most abundant isotope patterns with charge ≥ 2, selected with an isolation window of 2 *m/z* from the survey scan, fragmented by higher energy collisional dissociation (HCD) with normalized collision energies of 30 and dynamically excluded for 10 s. The maximum ion injection times for the survey scan and the MS/MS scans were 100 and 200 ms and the ion target values were set to 10^6^ and 10^5^, respectively.

#### MS/MS data processing

All data generated were searched using the Sequest HT search engine contained in the Thermo Scientific Proteome Discoverer software, version 2.1. The experimental MS/MS spectra were correlated to tryptic peptide sequences by comparison with the theoretical mass spectra obtained by in silico digestion of the UNIPROT *Mus musculus* proteome database (54,109 entries), downloaded in February 2019 (www.uniprot.org). The following criteria were used for the identification of peptide sequences and related proteins: trypsin as enzyme, three missed cleavages per peptide, mass tolerances of ± 10 ppm for precursor ions and ± 0.6 Da for fragment ions. Percolator node was used with a target-decoy strategy to give a final false discovery rates (FDR) at peptide spectrum match (PSM) level of 0.01 (strict) based on q-values, considering maximum deltaCN of 0.05 [56]. Only peptides with high confidence, minimum peptide length of six amino acids, and rank 1 were considered. Protein grouping and strict parsimony principle were applied.

#### Label-free differential analysis and hierarchical clustering

To improve the identification of differentially expressed proteins, a label-free approach based on spectral count (SpC) and linear discriminant analysis (LDA) was performed as previously reported [39]; Healthy (*n* = 6), hyalomatrix (*n* = 6), Angio^PRP^ (*n* = 6), PRP (*n* = 4) were considered. Specifically, proteins with *p* value (≤ 0.01), corresponding to *F* ratio ≥ 6, were retained and considered differentially expressed with high confidence. Pairwise comparisons (Healthy vs Hyalomatrix, Healthy vs Angio^PRP^ and Healthy vs PRP) were performed by Multidimensional Algorithm Protein Map (MAProMa) applying a threshold of 0.4 on Dave (Differential Average) MAProMa index [57]; DAve, which evaluates changes in protein expression, was defined as (*X − Y*)/(*X* + *Y*)/0.5, where *X* and *Y* terms represent the SpC of a given protein in two compared samples.

#### Network analysis

A protein–protein interaction (PPI) network was built by combining differentially expressed proteins (*n* = 254) and the *Mus Musculus* PPI network retrieved from Cytoscape StringApp [58]; only experimentally and database defined PPIs, with a score > 0.15 and > 0.3, respectively, were considered. The resulting sub-networks were visualized and analyzed by Cytoscape and its plugins, as previously reported [59]. Specifically, Cytoscape BingoApp [60] and Cytoscape StringApp [58] were used for evaluating the most represented GO terms; as for BingoApp, Mus musculus organism, hypergeometric test, Benjamini–Hochberg FDR correction and a significance level ≤ 0.01 were applied, while default parameters were used for StringApp.

#### Statistics

Sample size was determined considering a statistical test power of 0.80 and an alpha value of 0.05. Results indicated that a sample size of 15 animals (*n* = 5 per group) would enable to detect a minimum difference in protein expression of 0.35 with an expected standard deviation of 0.15. To detect outliers, Grubb’s test was applied for each parameter. A probability value < 0.05 was considered significant. All analyses were performed as previously described [55] using Sigma Stat 11.0 dedicated software (Systat Software Inc., San Jose, CA, USA). Identified proteins were evaluated by LDA (JMP15.2 software SAS; *F* ratio > 6 and a *p*-value < 0.01) and MAProMa platforms.

## Supplementary Information

Below is the link to the electronic supplementary material.

Supplementary file1 (DOC 78765 KB)

Supplementary file2 (XLSX 524 KB)

Supplementary file3 (XLS 119 KB)

## Data Availability

The datasets generated during and/or analysed during the current study are available from the corresponding author on reasonable request.
