# Supplementary material for: Effective high-throughput isolation of enriched platelets and circulating pro-angiogenic cells to accelerate skin-wound healing
Source: Cell Mol Life Sci. 2022 Apr 26;79(5):259. doi: 10.1007/s00018-022-04284-4 (PMC9042989; doi:10.1007/s00018-022-04284-4)
Supplement: Supplementary file 1 — Supplementary file1 (DOC 78765 KB) [file 18_2022_4284_MOESM1_ESM.doc]

**Supplementary Figures**

**

**

**Suppl. Fig. S1**

**Cytometry gate strategy**

**A** Gate strategy for cytometry analysis of Angio^PRP^ subpopulations: T lymphocytes (Syto16+/CD45+/CD3+/CD19-), B lymphocytes (Syto16+/CD45+/CD3-/CD19+), monocytes (Syto16+/CD45+/CD3-/CD14+), granulocytes (Syto16+/CD45+/CD3-/CD14+/CD15+) and NKs (Syto16+/CD45+/CD3-/CD16+/CD56+). **B** Gate strategy for cytometry analysis and sorting of Tang (Syto16+/CD45+/CD3+/CD31+/CD184+) and EPC subpopulations (Syto16+/CD45+/CD31+/CD90+/CD146+).

**Suppl. Fig. S2**

**Histological characterization of healthy and PBS treated skin.**

**A, B** Hematoxylin and eosin histological reconstruction of healthy skin (**A**) and saline solution-treated skin (**B**) sections 21 DPI (scale bar = 500µm, magnification scale bar = 200 µm). Immunofluorescence reconstructed images of cytokeratin 5 (CK5) staining for basal layer identification (central panels, scale bar = 250µm) and cytokeratin 10 (CK10) and β-catenin staining for dermal-epidermal junction (right panels, scale bar = 250µm). Frame magnifications are reported in the enlarged images (scale bar = 200µm). Orcein staining of healthy skin section and saline solution treated skin (lower panels, scale bar = 500µm, magnification scale bar = 200 µm). Masson’s trichrome staining (scale bar = 500 µm) and collagen VI immunofluorescent staining for healthy and saline solution treated skin (scale bar = 75µm).

**
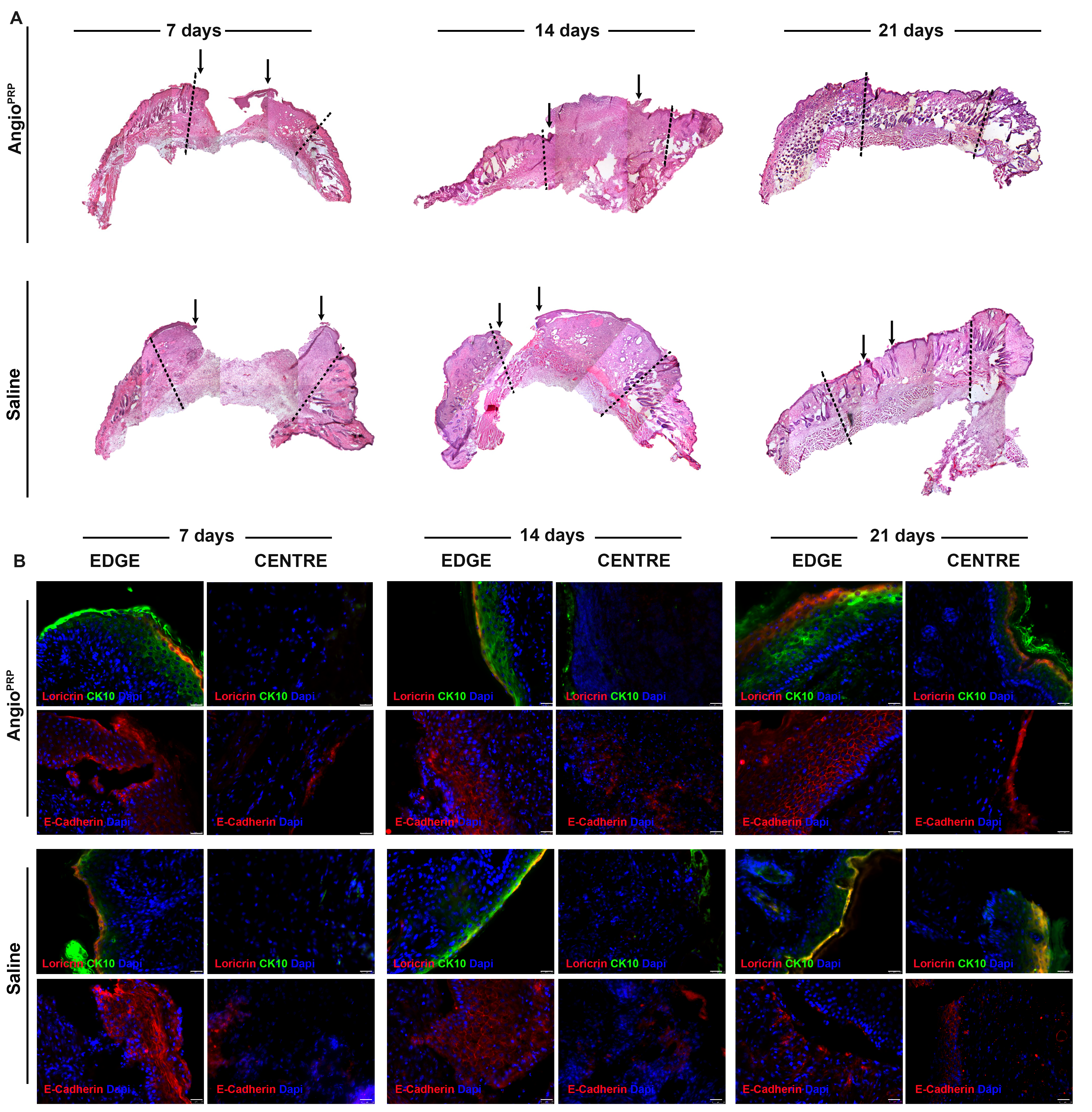
**

**Suppl. Fig. S3**

**Histological and immunofluorescence characterization of wound healing progression**

**A** Hematoxylin and eosin histological reconstruction of wounded skin samples, treated with Angio^PRP^ or saline solution at 7, 14 and 21 DPI; dashed lines represent the original wound boundary (5 mm-diameter excision); arrows indicate the re-epithelialization edges. **B** Immunofluorescence staining for loricrin, cytokeratin (CK10) and E-cadherin was performed to evaluate re-epithelialization and dermal-epidermal junction at 7, 14 and 21 DPI; images show the edge and the center of wounded area (scale bar = 25µm)


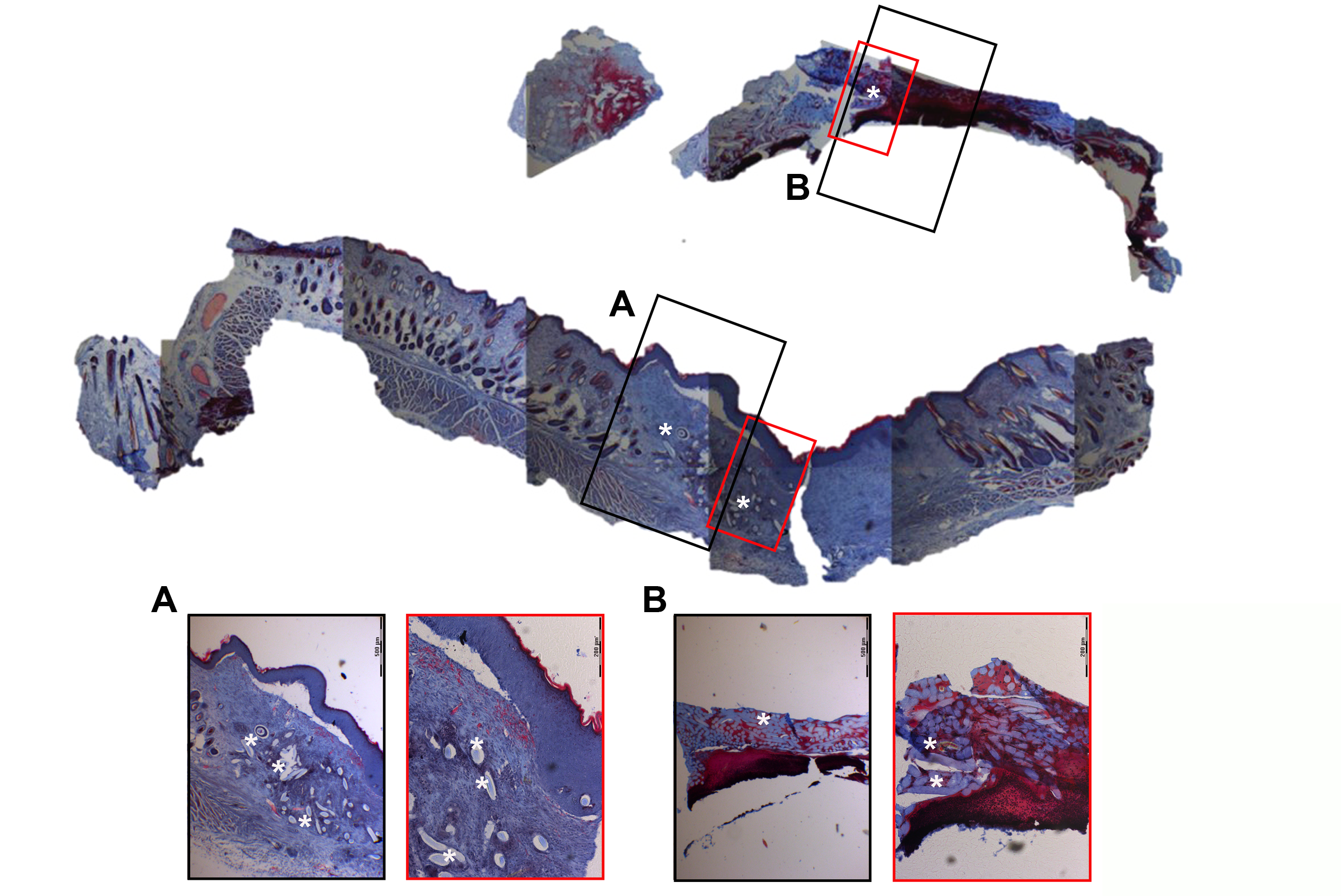


**Suppl. Fig. S4**

**Material inclusions in Hyalomatrix treated wounds**

Masson’s trichrome histological reconstruction of skin sample treated with Hyalomatrix 21 DPI. Lower panels report frame magnifications of A and B; small fibers of Hyalomatrix component are pointed out to indicate the inclusion of exogenous material in wound bed during the healing process (black frames scale bar = 500 µm; red frames scale bar = 200 µm).
